# Endoscopic ultrasonography with fine-needle aspiration for histological diagnosis of solid pancreatic masses: a meta-analysis of diagnostic accuracy studies

**DOI:** 10.1186/s12876-016-0519-z

**Published:** 2016-08-31

**Authors:** Omar Banafea, Fabian Pius Mghanga, Jinfang Zhao, Ruifeng Zhao, Liangru Zhu

**Affiliations:** 1 Division of Gastroenterology, Union Hospital, Tongji Medical College, Huazhong University of Science and Technology, No. 1277 Jiefang Avenue, Wuhan, 430022 Hubei Province China; 2 Department of Nuclear Medicine, Union Hospital, Tongji Medical College, Huazhong University of Science and Technology, Hubei Province Key Laboratory of Molecular Imaging, Wuhan, 430022 China

**Keywords:** Endoscopic ultrasound, Fine needle aspiration, Pancreatic mass

## Abstract

**Background:**

Previous studies have demonstrated that endoscopic ultrasound-fine needle aspiration (EUS-FNA) is a reliable tool for diagnosing pancreatic lesions; however, the reported sensitivity and specificity vary greatly across studies. The aim of this study was to pool the existing literature and assess the overall performance of EUS-FNA in the diagnosis of solid pancreatic lesions.

**Methods:**

A systematic search of MEDLINE, Cochrane Database for Systematic Reviews, and EMBASE was performed to identify original and review articles published between January 1995 and January 2014 that reported the accuracy of EUS-FNA in the diagnosis of pancreatic masses. Quality of the included studies was assessed using the quality assessment of diagnosis accuracy studies score tool. Meta-DiSc software was used to calculate the pooled sensitivity and specificity, positive and negative likelihood ratios, and to construct the summary receiver operating characteristics curve.

**Results:**

Twenty studies involving a total of 2,761 patients were included in the study. The pooled sensitivity and specificity of EUS-FNA in the diagnosis of solid pancreatic lesions were 90.8 % [95 % confidence interval (CI), 89.4–92 %] and 96.5 % (95 % CI, 94.8–97.7 %), respectively. The positive and negative likelihood ratios were 14.8 (95 % CI, 8.0–27.3) and 0.12 (95 % CI, 0.09–0.16), respectively. The overall diagnostic accuracy was 91.0 %.

**Conclusions:**

Our findings suggest that EUS-FNA has high sensitivity and specificity in the diagnosis of solid pancreatic lesions.

## Background

Pancreatic cancer is the tenth most common type of cancer, and the fourth leading cause of cancer-related deaths among men and women, accounting for 6 % of all cancer-related deaths worldwide [1]. Pancreatic cancer is difficult to diagnose in its early stages, and nearly 26 % of all diagnosed cases have regional spread, with 52 % of cases reported to have metastatic disease at the time of diagnosis [2]. Studies have shown that one-year survival rate for pancreatic cancer is 24 %, and the overall 5-year survival rate is 5 % [2]. Since curative resection is currently the only potential cure for patients with pancreatic cancer, early diagnosis has an important impact on prognosis.

Pancreatic lesions encompass a variety of benign and malignant conditions, and the diagnosis of pancreatic cancer is complicated by indistinct detection of pancreatic masses either clinically or by imaging. Clinically, the diagnosis of a regional pancreatic mass may be confused with that of a primary pancreatic tumor, as in pancreatic adenocarcinoma, while focal chronic pancreatitis may be confused with pancreatic metastasis from a distant primary tumor [3]. Thus, accurate preoperative diagnosis is essential for selecting an appropriate treatment for these lesions [4].

Currently, there are many laboratory tests and imaging techniques that may be useful in discriminating pancreatic lesions [5, 6]. Among them, cytological examination of pancreatic masses by fine needle aspiration (FNA) can assist greatly in differentiating a pancreatic tumor from other malignancies. Endoscopic ultrasound (EUS), due to its high resolution, can provide easy visualization of the pancreas, common bile duct and adjacent anatomic structures, and has been the most important imaging modality for the diagnosis of pancreatic tumors [7]. EUS combined with FNA (EUS-FNA) has been demonstrated to be more accurate in diagnosing solid pancreatic lesions and has gained wide acceptance [7]. However, the reported sensitivity and specificity of EUS-FNA vary greatly across studies (sensitivity: 73.20–96.50 %; specificity: 71.40–100 %) [7–26]. In addition, the majority of previous studies were dependent on single-center trials. Since there is a learning curve for FNA, its diagnostic accuracy is greatly influenced by operator experience [22]. In addition, the performance of FNA may be related to the size and location of pancreatic lesions and the presence of an on-site cytopathologist [7, 26]. At present, there has been no systematic approach to estimate the accuracy of EUS-FNA in diagnosing solid pancreatic lesions.

The current meta-analysis aimed at reviewing the existing literature and evaluating the overall performance of EUS-FNA in diagnosing solid pancreatic lesions.

## Methods

### Identification of studies

A systematic search of PubMed (including MEDLINE compiled by the United States National Library of Medicine, Bethesda, Maryland, USA), EMBASE (Elsevier, Amsterdam, Netherlands), and the Cochrane Database for Systematic Reviews (The Cochrane Collaboration, Oxford, UK) was performed to identify published original and review articles reporting the accuracy of EUS-FNA in the diagnosis of pancreatic masses. The electronic search was supplemented by a manual search of the listed references. Searches were limited to studies conducted from January 1995 to January 2014. We used the keywords [“pancreatic mass” or “pancreatic lesion” or “pancreatic tumor”] and [“endoscopic ultrasound” or “endoscopic ultrasound fine needle aspiration” or “EUS-FNA” or “EUS-FNA in pancreatic lesions”)] and [“sensitivity” or “specificity” or “diagnostic accuracy”].

We identified 285 studies through this search strategy. We also hand-searched several imaging and oncology journals for the specified period to ensure that the electronic search did not miss reports of eligible studies; no additional study was identified using this strategy.

The reference list of the retrieved studies was searched for any additional publications, and none of the articles was found in this approach. We restricted searching to studies that were published in English only and included more than 10 patients.

### Eligibility criteria

Studies were eligible for inclusion if they fulfilled the following criteria: (i) articles were published in English; (ii) appropriate data were presented to enable computation of true positive (TP), false negative (FN), false positive (FP) and true negative (TN) results of EUS-FNA in the diagnosis of solid pancreatic lesions; (iii) at least 10 patients and/or lesions were included; (iv) a final diagnosis was obtained by surgical biopsy or histological examination of surgically resected specimen; (v) the population had a suspected solid pancreatic mass based on imaging modalities such as ultrasound, EUS, computed tomography (CT) and magnetic resonance imaging (MRI), and only patients who had a solid pancreatic mass (in case of mixed lesions, separate results were reported for solid and cystic lesions) were included in the study; (vi) studies were retrospective and/or prospective, and had results of EUS-FNA based on surgical cytological/ histological specimens, or a follow-up period of at least 6 months; and (vii) articles were published from January1995 to January 2014.

We excluded: (i) case reports and abstracts; (ii) studies that did not report sufficient data to construct a diagnostic 2 × 2 contingency table to calculate statistics including TP, FP, TN and FN; and (iii) studies involving patients with a cystic lesion or other malignancy like cholangiocarcinoma, duodenal adenocarcinoma, and periampullary adenocarcinoma and studies that involved other FNA procedures like CT-guided FNA or MRI-guided FNA. We excluded cystic lesions from the current analysis, because their diagnosis and management were different from those of solid pancreatic lesions [27].

### Study quality assessment

We used the quality assessment of diagnosis accuracy studies (QUADAS) tool [28] to assess the methodological quality of the included studies. The tool had 14 questions with responses denoted as “yes,” “no,” or “unavailable.” A score of 1 was given to a “yes” response, and a score of zero was given if the response was “no” or “unavailable”. An article was deemed of adequate quality for inclusion if it scored a minimum of 8 of 14 points in the “QUADAS” checklist as in Table 1.

**Table 1:** The characteristics of included studies in current meta-analysis

Study name	QUADAS score (14)	Study design	Study center	On site Cyto.
Giovannini et al. 1995 [8]	12	R	S	No
Cahn et al. 1996 [9]	8	R	M	No
Bhutani et al. 1997 [10]	10	p	S	Yes
Faigel et al. 1997 [11]	12	P	S	Yes
Chang et al. 1997 [23]	11	P	S	No
Bentz et al. 1998 [24]	13	P	S	Yes
Voss et al. 2000 [12]	12	P	S	No
Gress et al. 2001 [13]	11	p	S	No
Ylagan et al. 2002 [14]	10	R	S	No
Harewood 2002 [15]	13	P	M	Yes
Raut et al. 2003 [16]	13	P	S	No
Afify et al. 2003 [17]	11	R	S	Yes
Agarwal et al. 2004 [18]	11	R	S	No
Ryozawa et al. 2005 [19]	9	R	M	Yes
Eloubeidi et al. 2007 [20]	13	P	S	No
Fisher et al. 2009 [25]	13	P	S	No
Krishna et al. 2009 [21]	12	P	S	Yes
Touchefeu et al. 2009 [22]	13	P	S	No
Cherian et al. 2010 [7]	10	P	S	No
Uehara et al. 2011 [26]	9	R	S	No

*P* Prospective, *R* Retrospective, *M* Multiple centers, *S* Single center

### Data extraction

Data from all eligible studies were extracted independently by two of the authors (O.B and F.P.M). Information extracted included the first author’s name, journal, year of publication, study design, sample size and clinical indication. We also extracted demographic characteristics including mean or median age, patient gender, number of lesions and lesion size. Other extracted information included needle manufacturing company, frequency of EUS, needle size and number of needles that passed through the lesion during procedure. In cases of any differences between the two authors, a consensus was reached by discussion.

### Statistical analysis

For each included study, we constructed a 2 × 2 table to calculate the TP, FN, FP and TN values. The data were then analyzed using Meta-DiSc software (version 1.4; Unit of Clinical Biostatistics Team of the Romany Cajal Hospital, Madrid, Spain) to compute sensitivity, specificity, positive likelihood ratio, negative likelihood ratio, positive predictive value (PPV), negative predictive value (NPV) and diagnostic odds ratio (DOR) for each study. As per the DerSimonian-Liard random effects model, we pooled all results and using the same model, we constructed a summary receiver operating characteristic curve (SROC). By numeric integration of the SROC using the trapezoidal equation, the software was used to compute the area under the curve (AUC). A preferred test has an AUC close to 1, and an AUC close to 0.5 is considered a poor test. NPV and FP rate (i.e., 1 – specificity) were also calculated. Q*, the maximum joint specificity and sensitivity, was calculated from the SROC. This is the point on the SROC curve where sensitivity is equal to specificity. A chi-square test was used to test for the occurrence of heterogeneity among studies, and sources of heterogeneity were explored using meta-regression analysis. *P-*values < 0.05 were considered statistically significant.

## Results

### Eligible studies

**Fig. 1:**
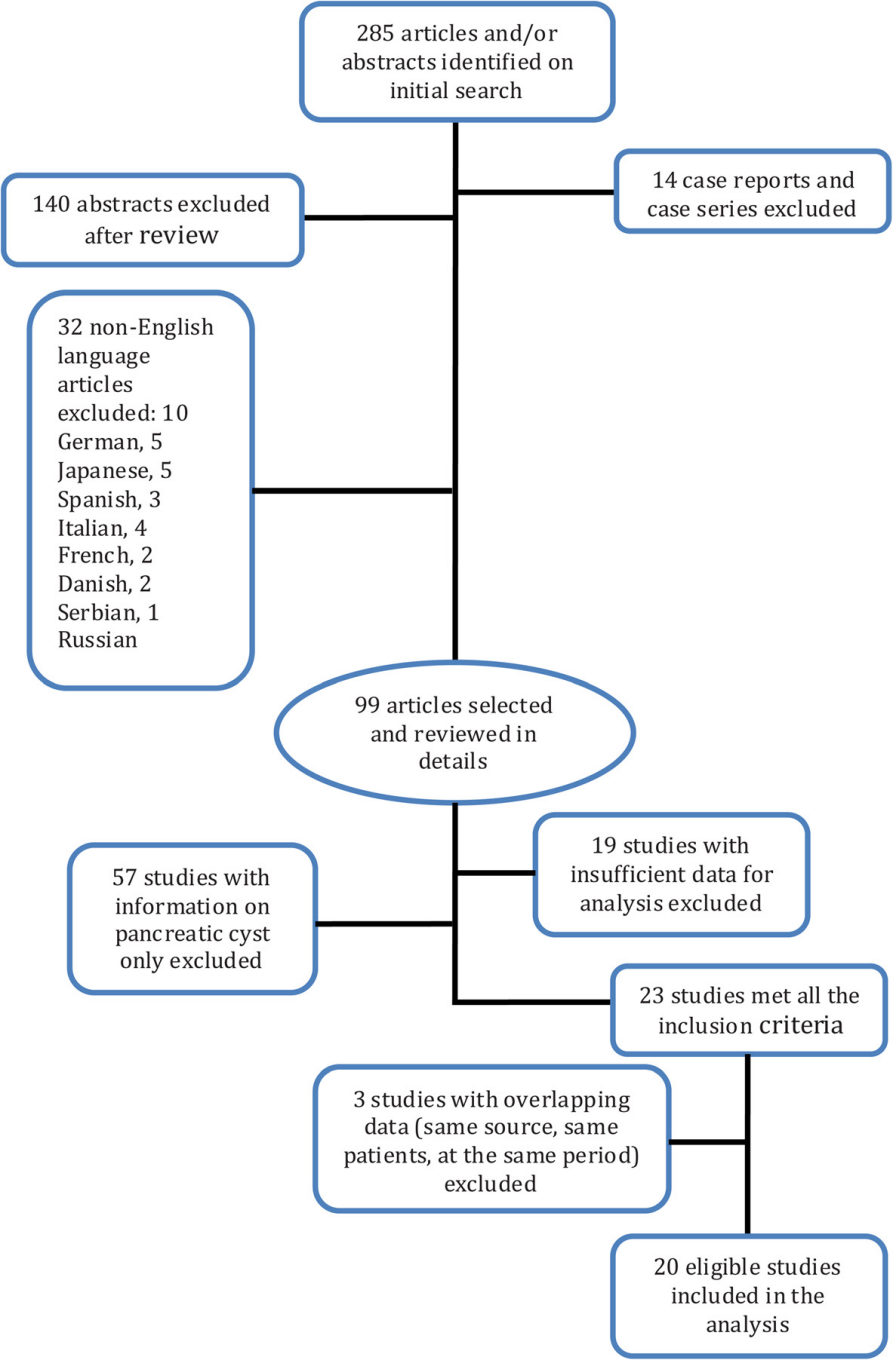
Flow diagram of the study selection process

Our searches yielded a total of 285 titles and abstracts. Of these, 140 abstracts and 32 studies published in languages other than English (10 in German, 5 in Japanese, 5 in Spanish, 4 in French, 3 in Italian, 2 in Danish, 2 in Serbian and 1 in Russian) were excluded. Upon further review of the studies, 14 case reports and case series with a sample size less than 10 patients were excluded. We also excluded 57 studies with information on pancreatic cyst only and 19 studies with insufficient data. Five duplicate studies, three in one batch and two in the other, had the same sources of data, so we excluded the 3 studies from the duplicated studies and included the 2 studies that had included data from the same database (Table 3). Finally, a total of 20 studies [7–26] were eligible for analysis based on the inclusion and exclusion criteria. Figure 1 shows the flow chart of study selection.

### Study description and patient characteristics

Of the 20 studies included, 13 were prospective [7, 10–13, 15, 16, 19, 20–25] and 7 were retrospective [8, 9, 14, 17–19, 26] (Table 1). The studies involved a total of 2,761 patients with a total of 2,776 pancreatic lesions. The median age of the subjects was reported in 15 articles, and 5 did not mention the age of the patients. The male/female ratio of the study subjects was 1.3:1.

### EUS-FNA techniques

The majority of studies used 22-gauge needles in EUS-FNA procedures [7, 11, 13–22, 24–26], although some of these needles were made by different manufacturers. Other sizes used were 19-gauge (Wilson cook, Winston Salem, North Carolina NC) [10, 13] and 25-gauge (manufacturing company not mentioned) needles [8].

Fifteen of the 20 included studies mentioned pancreatic mass size which varied between 0.6 and 14 cm whereas the rest 5 studies did not mention the size of masses [7, 9–13, 15, 18–20, 22–26]. The mean tumor size was 3.4 cm, with a range of 0.6–14 cm. The median number of needle passes through each pancreatic lesion was 3.4, ranging between 1 and 5.

### Safety of EUS-FNA

As shown in Table 2, actual complications of EUS-FNA procedures occurred in 35 of 1,760 patients in 15 studies that mentioned complications [7, 8, 10–13, 15, 16, 19, 20, 22, 23, 25, 26], mainly abdominal pain, pancreatitis, hematoma, bleeding at needle sites and fever (not accompanied by other symptoms). There were two cases of major complication (duodenal perforations, which were immediately managed by laparotomy).

**Table 2:** Complications of EUS-FNA reported in the included studies

Study	Number	Percent	Post-procedural complications
Giovannini et al. 1995 [8]	0	0	No complications reported
Cahn et al. 1996 [9]	Unknown	-	-
Bhutani et al. 1997 [10]	1/47	2	Infection (*n* = 1)
Faigel et al. 1997 [11]	0/45	0	No complications reported
Chang et al. 1997 [23]	1/44	2	Fever (*n* = 1)
Bentz et al. 1998 [24]	Unknown	-	-
Voss et al. 2000 [12]	5/90	5	Bleeding (*n* = 4), abdominal pain (*n* = 1)
Gress et al. 2001 [13]	3/102	2.9	Gastric mucosal bleeding (*n* = 2), pancreatitis (*n* = 1)
Ylagan et al. 2002 [14]	1/91	1	Acute pancreatitis (*n* = 1).
Harewood 2002 [15]	1/185	0.5	Mild pancreatitis (*n* = 1).
Raut et al. 2003 [16]	4/233	2	Duodenal perforation (*n* = 2), abdominal pain (*n* = 1), pancreatitis (*n* = 1)
Afify et al. 2003 [17]	Unknown	-	-
Agarwal et al. 2004 [18]	2/81	2.5	Abdominal pain (*n* = 2)
Ryozawa et al. 2005 [19]	0	0	No complications reported
Eloubeidi et al. 2007 [20]	11/547	2	Acute pancreatitis (*n* = 5), abdominal pain (*n* = 3), fever (*n* = 2), the use of reversal medication (*n* = 1)
Fisher et al. 2009 [25]	2/100	2	Mucosal bleeding (*n* = 1), abdominal pain (*n* = 1)
Krishna et al. 2009 [21]	Unknown	-	-
Touchefeu et al. 2009 [22]	2/90	2.2	Fever (*n* = 1), abdominal pain (*n* = 1)
Cherian et al. 2010 [7]	Unknown	-	-
Uehara et al. 2011 [26]	2/120	1.6	Mild pancreatitis (*n* = 2)

### Pooled results

**Fig. 2:**
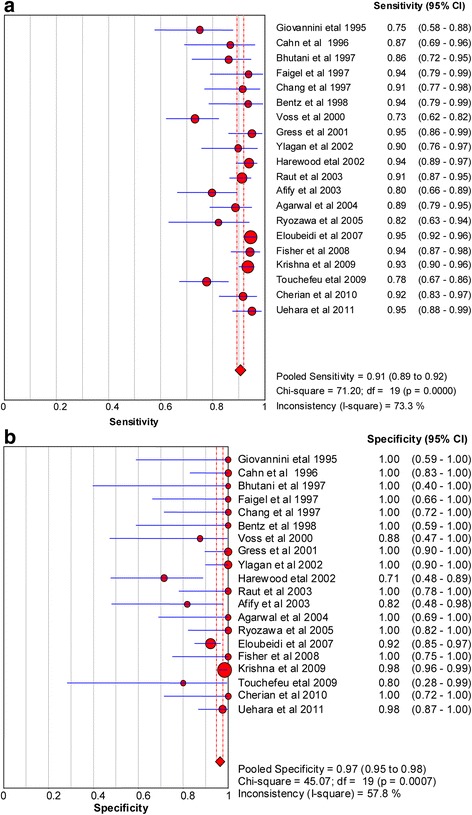
Forest plots of sensitivity (**a**) and specificity (**b**)

**Fig. 3:**
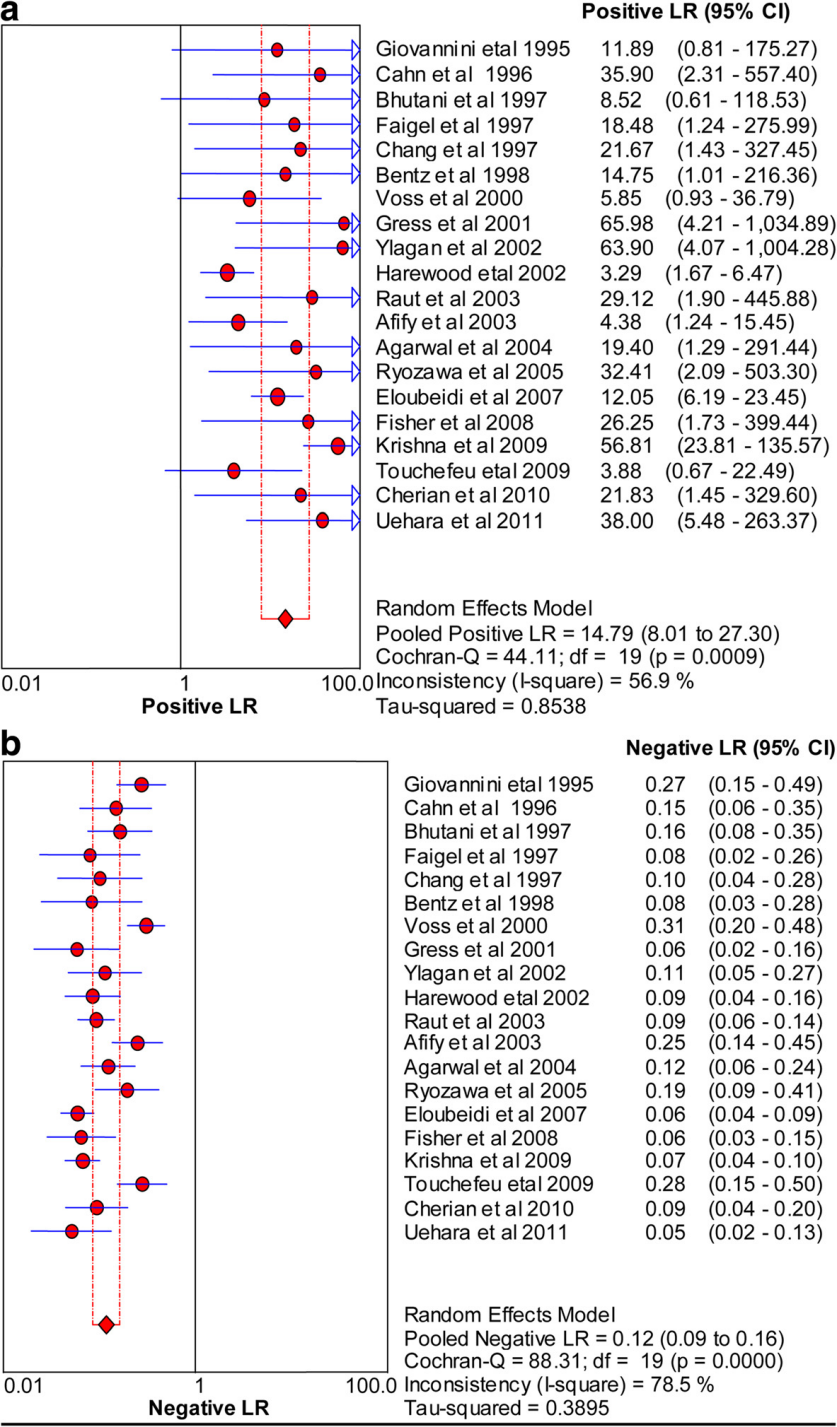
Forest plots of positive likelihood ratio (**a**) and negative likelihood ratio (**b**)

**Fig. 4:**
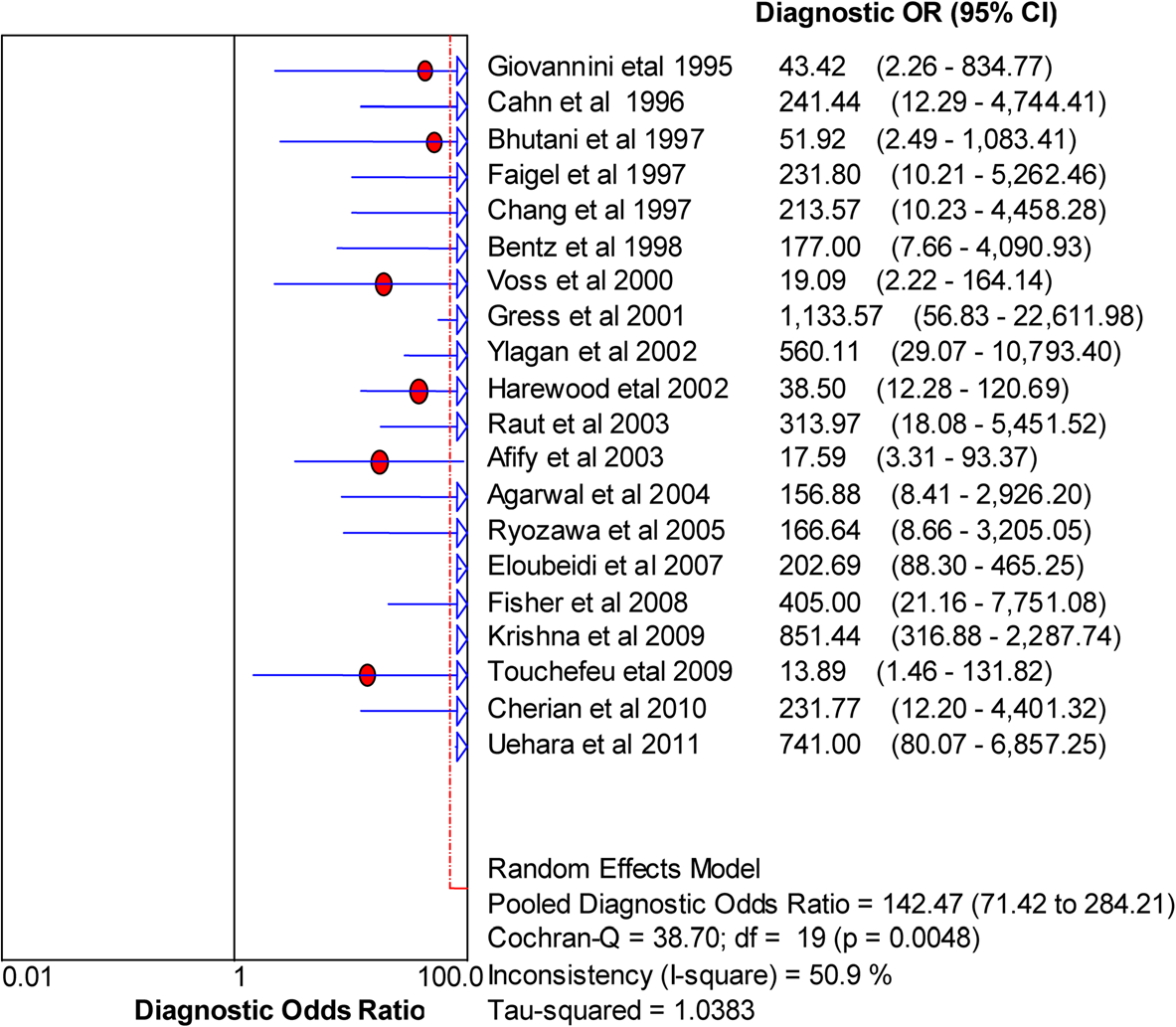
Forest plot of diagnostic odds ratio

The sensitivity and specificity of EUS-FNA in the diagnosis of solid pancreatic masses were found to range from 73.20 to 96.50 % and 71.40 to 100 %, respectively. The median sensitivity and specificity were 91.30 % and 100 %, respectively. The pooled sensitivity and specificity were 90.80 % [95 % confidence interval (CI): 89.40–92.00 %] and 96.5 % (95%CI: 94.8–7.7 %), respectively (Fig. 2). The positive and negative likelihood ratios were 14.80 (95%CI, 8.00–27.30) and 0.12 (95%CI, 0.09–0.16), respectively (Fig. 3). The diagnostic odds ratio is 142.47 (71.42–284.21) (Fig. 4).

### ROC analysis

**Fig. 5:**
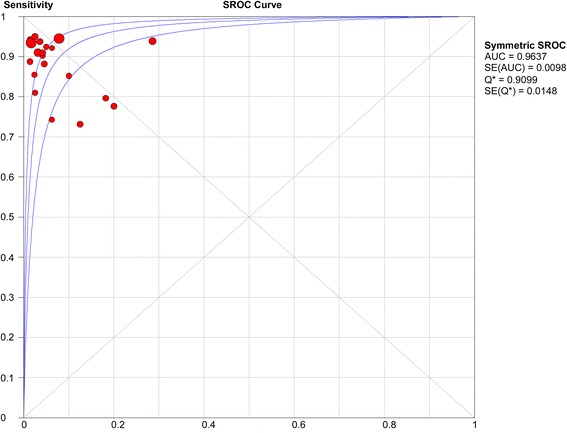
Summary receiver operating characteristic (SROC) curve analysis

The AUC was used to summarize the overall diagnostic accuracy of EUS-FNA. From the curve, the maximum joint sensitivity and specificity, denoted as Q* (the point at which the sensitivity and specificity of a diagnostic tool are equal), was found to be 91.0 % (Fig. 5). This finding suggests a relatively high overall diagnostic performance of EUS-FNA in the diagnosis of solid pancreatic lesions.

### Sources of heterogeneity

To explore potential sources of heterogeneity, a meta-regression analysis was performed. A multivariable regression model with a backward stepwise algorithm was used. The possible sources of heterogeneity in our study included publication bias, study design and methodological quality. None of the analyzed variables showed statistical significance (Table 3), e.g*.*, higher quality studies (QUADAS score ≥ 10) and lower quality studies (QUADAS score <10) were not significantly different.

**Table 3:** Summary of diagnostic performance of endoscopic ultrasonography with fine-needle aspiration for solid pancreatic lesions

Study	Year	TP	FP	FN	TN	Sensitivity	Specificity	LR+	LR-	DOR
Giovannini	1995	27	0	9	7	75 (58–88)	100 (59–100)	11.9 (0.81–175)	0.3 (0.15–0.49)	43 (2–835)
Cahn	1996	26	0	4	20	87 (69–96)	100 (83–100)	35.9 (2.3–557)	0.15 (0.06–0.4)	241 (12–4744)
Bhutani	1997	37	0	6	4	86 (72–94)	100 (40–100)	8.5 (0.6–118.5)	0.16 (0.08–0.35)	52 (2.5–1083)
Faigel	1997	30	0	2	9	94 (79–99)	100 (66–100)	18.5(1.2–276)	0.08 (0.02–0.27)	232(10–5263)
Chang	1997	32	0	3	11	91 (77–98)	100 (72–100)	21.7 (1.4–327.5)	0.1 (0.04–0.28)	214(10–4458)
Bentz	1998	29	0	2	7	94 (79–99)	100 (59–100)	14.8 (1–216.4)	0.08 (0.025–0.28)	177(8–4090)
Voss	2000	60	1	22	7	73 (62–82)	88 (47–99.7)	5.8 (0.9–36.8)	0.3 (0.2–0.48)	19 (2.2–164)
Gress	2001	57	0	3	34	95 (86–99)	100 (90–100)	66 (4.2–1035)	0.06 (0.02–0.16)	1134 (56.828–22612)
Ylagan	2002	35	0	4	35	90 (76–97)	100 (90–100)	63.9 (4.1–1004)	0.11 (0.048–0.27)	560(29–10793)
Harewood	2002	154	6	10	15	94 (89–97)	71 (48–89)	3.3 (1.7–6.5)	0.09 (0.044–0.17)	39(12–121)
Raut	2003	197	0	19	15	91 (87–95)	100 (78–100)	29 (1.9–446)	0.09 (0.06–0.14)	314(18.083–5451.5)
Afify	2003	43	2	11	9	80 (67–89)	82 (48–98)	4.4 (1.2–15.5)	0.25 (0.14–0.45)	18 (3–93)
Agarwal	2004	63	0	8	10	89 (79–95)	100 (69–100)	19 (1.3–291)	0.12 (0.07–0.24)	157 (8–2926.2)
Ryozawa	2005	23	0	5	19	82 (63–94)	100 (82–100)	32.4 (2.1–503)	0.2 (0.09–0.414)	166 (9–3205)
Eloubeidi	2007	414	8	24	94	95 (92–97)	92 (85–97)	12.1 (6.2–23.5)	0.06 (0.04–0.09)	202.69 88.302–465.25
Fisher	2009	82	0	3	13	97 (90–99)	100 (75–100)	26.3 (1.7–399)	0.07 (0.03–0.15)	405 (21.2–7751)
Krishna	2009	299	5	21	299	93 (90–96)	98 (96–99.5)	57 (24–136)	0.07 (0.044–0.1)	851 (317–2288
Touchefeu	2009	66	1	19	4	78 (67–86)	80 (28–99.5)	3.9 (0.7–22.5)	0.28 (0.16–0.5]	13.9 (1.5–132)
Cherian	2010	65	0	6	11	92 (83–97)	100 (72–100)	22 (2–330)	0.09 (0.05–0.2)	232 (12–4401)
Uehara	2011	76	1	4	39	95 (88–99)	98 (87–100)	38 (6–263)	0.05 (0.02–0.13]	741 (80–6857)

*TP* true positive, *FP* false positive, *FN* false negative, *TN* true negative, *LR+* positive likely ratio, *LR*- negative likely ratio, *DOR* Diagnostic Odds Ratio

## Discussion

Many previous studies have demonstrated that EUS-FNA is a reliable tool for the diagnosis of pancreatic masses; however, the reported sensitivity and specificity varied greatly among these different studies [7–26]. In this study, we conducted a meta-analysis to pool the existing literature and assess the overall performance of EUS-FNA in the diagnosis of solid pancreatic lesions. We found that the pooled sensitivity and specificity were as high as 90.8 % and 96.5 %, respectively. The SROC analysis revealed that the Q* value, which represents the maximum joint sensitivity and specificity, was 91 %. These findings suggest that EUS-FNA has a high accuracy in diagnosing solid pancreatic lesions.

The wide variation of the reported sensitivity and specificity of EUS-FNA may be due to a combination of several factors. Operator experience has been proposed as one of the most significant factors that affect the accuracy of EUS-FNA [7, 26, 29], which is a demonstrated significant predictor of diagnostic accuracy in pancreatic lesions in the multivariable model [30]. Since the majority of previous studies depended on the results of one center and the operators had greatly varied experience, the diagnostic performance of EUS-FNA may have been overestimated or underestimated. Thus, an important strength of our meta-analysis is that it included studies performed by operators with different expertise from different centers in different countries. The level of operators’ experience should be reported in future studies. In addition, the diagnostic accuracy of EUS-FNA for pancreatic lesions may also be affected by tumor size and location, needle size, and the presence of an on-site cytopathologist [26, 31], although some studies reported that the diagnostic accuracy of EUS-FNA is irrespective of these parameters [26]. Future studies should carefully resolve these problems.

Low NPV has been suggested to be a major drawback of EUS-FNA, and this may limit its clinical utility in patients with suspected pancreatic cancer because early resectable tumors may be missed [21]. An NLR < 0.1 often suggests that the predictive value of a given diagnostic tool is valid or rather convincing. In the present study, the pooled NLR was 0.118 (95%CI: 0.086–0.163), which is near 0.1. Considering that we calculated all inadequate biopsies and technical failures in the included studies as FNs, the pooled NLR may have been underestimated. In this regard, the predictive value of EUS-FNA for solid pancreatic lesions is acceptable.

The FN results of EUS-FNA in the diagnosis of pancreatic masses are often caused by a high frequency of inadequate specimens due to several reasons. First, comparative studies of EUS-FNA have demonstrated that larger needles were associated with inferior accuracy rates because of their disadvantage in placement precision in pancreatic lesions located in difficult anatomical positions [32–35]. Second, the number of needle passes through the lesion may affect the collection of adequate specimens [35], and a recent report recommended that seven needle passes would be necessary to ensure a highly accurate diagnosis of solid pancreatic lesions by EUS-FNA [36]. Finally, the absence of on-site cytology in the procedure may also reduce the aspiration of adequate sample, although some studies indicated that the unavailability of a cytopathologist did not significantly affect the diagnostic yield [26, 37]. The current study indicated that the absence of on-site cytology has no significant impact on the diagnostic yield (Table 4).

**Table 4:** Diagnostic accuracy of all 20 included studies

	No. of studies	Sensitivity	Specificity	LR(+)	LR(-)	DOR
All of 20 studies		90.80	96.5	14.80	0.12	142.47
	20					
*P*		0	.0007	0.0009	0.000	0.0048
Study design:						
Prospective	13	91.4	94.3	10.928	0.110	122.14
*P*						
		0	0.006	0.130	0	0.348
Study design:						
Retrospective	7	89.5	97.9	19.004	0.128	162.38
*P*		0.001	0.076	0.009	0	0.001
QUADAS score ≥10		90.8	96.2	13.162	0.116	127.44
*P*	17					
		0	0	0.001	0	0.002
QUADAS score <10		90.6	98.7	36.008	0.119	370.78
	3					
*P*		0.103	0.503	0.996	0.076	0.687
With on-site cytology.		91.5	96.5	12.136	0.120	108.83
	7					
*P*		0.024	0	0	0.002	0
Without on-site cytology.		904	96.5	14.365	0.114	170.05
	13					
*P*		0	0.097	0.757	0	0.263

Studies have indicated that EUS-guided FNA biopsy of pancreatic masses is as accurate as CT/US-guided and surgical biopsies [38]. However, EUS-FNA has many advantages over other techniques [39]: (i) the capability to get a sample from a tiny lesion; (ii) the capability to get a sample of the lesion through a part of the intestinal wall and decrease the risk of needle tract seeding; and (iii) the capability to supply extra information about staging of the disease. In particular, EUS-guided FNA can be carried out in the entire pancreas (the hook and tail included) [16, 17], even for difficult or unreachable regions via the percutaneous access or when the percutaneous route is not indicated [12].

Although many diagnostic modalities are currently available for the diagnosis of pancreatic lesions, some are associated with a higher incidence of complications. For example, the rate of complications of CT/US-guided FNA is as high as about 5 %, with pancreatitis being the most common complication [39]. In the present study, only 2.2 % (39/1760) of cases developed complications, and the majority of complications were mild, self-limited and seldom required transfusion, except that two cases had duodenal perforations that were successfully treated by surgery. These finding suggest that EUS-FNA is a safe technique for the diagnosis of solid pancreatic lesions.

Ultimately in considering to tumor size of pancreatic malignancy was diagnosed by Endoscopic Ultrasound-guided FNA with high accuracy regardless of their tumor size, or location. So we can get initial diagnosis of malignant lesion was obtained by EUS-guided FNA in all adenocarcinoma ≤ 10 mm but unfortunately most of studies included in this study mentioned only the range or median of the tumors size that included sizes < 2 cm which means that the small size (<2 cm) can be detected by EUS-FNA even if it cannot be detected by CT or US guided FNA.

## Conclusion

This meta-analysis indicates that EUS-FNA for pancreatic masses has a high overall sensitivity (91 %) and specificity (96.5 %). Compared with other diagnostic modalities, EUS-FNA may be safer and probably provides advantages over other modalities, such as the ability to detect a tiny lesion and obtain much more diagnostic information.

## Abbreviations

AUC: Area under the curve
CT: Computed tomography
DOR: Diagnostic odd ratio
ERCP: Endoscopic retrograde chlolaniopancreatography
EUS: Endoscopic ultrasound
EUS-FNA: Endoscopic ultrasound with fine needle aspiration
FN: False negative
FP: False positive
MRI: Magnetic resonance imaging
NLRs: Negative likelihood ratio
PC: Pancreatic cancer
PLRs: Positive likelihood ratio
ROC: Receiver operating characteristic curve
ROSE: Rapid on-site evaluation
SROC: Summary receiver operating characteristic curve
TN: True negative
TP: True positive
